# Current News

**Published:** 1892-07

**Authors:** 


### Current News.

### THE WILCOX BILL.

TO THE SENATORS AND REPRESENTATIVES OF MARYLAND, AND OTHER STATES, IN CONGRESS ASSEMBLED.

GENTLEMEN,—At the last Annual Meeting of the Maryland State Dental Society, held in Baltimore, on May 9 and 10, the following resolution was unanimously adopted :

"WHEREAS, A bill has been introduced in Congress, which provides that any person who shall neglect or refuse to give information to census officers shall be subject to a fine, 'not exceeding ten thousand dollars, to which may be added imprisonment for a period not exceeding one year,' and WHEREAS, the public press reports that the bill referred to 'will be used in the nature of thumbscrews and spiked boots to enforce replies,' and further, that 'the dentists of Baltimore, who by their refusal to answer certain questions . . . will be the first victims of the inquisitorial machine if the bill is passed ;' therefore, be it

"*Resolved*, That this Association reaffirms the resolution unanimously passed November, 1890, protesting against the action of the Census Bureau, in classifying dentists as manufacturers and refusing to fill out blanks.

"*Resolved, further*, That the chairmen of the several standing committees, as now constituted, be appointed to convey the sentiments of this Association to the Senators and Representatives of Maryland in Congress, and urge them to have the bill referred to amended, so as to omit dentistry, in all its branches, from the tabulations of the census."

We, therefore, the undersigned, the committee appointed in accordance with this resolution, respectfully submit the following to your consideration :

1. Dentistry is recognized as a profession by law. Dentists are not required to serve on juries; they have been recognized as professional men,—to wit, in the case of Noble, before Judge McArthur, of Washington city. The dentists of Baltimore have always been excused from jury duty.

2. Nearly all States of the Union have passed laws recognizing them as professional men, and in some of the States license fees are demanded from both physicians and dentists for the privilege of practising, and both are equally amenable to the law of malpractice.

3. Most of the States have appointed examining boards, to test the fitness of candidates for practice; similar laws are made bearing on the medical profession.

4. Maryland, Pennsylvania, New York, Massachusetts, Michigan, Minnesota, Kansas, Iowa, Illinois, Indiana, Missouri, Tennessee, Georgia, and Louisiana, have, through their respective Legislatures, granted charters to dental colleges, giving them authority to educate students in dentistry, and to confer upon those properly qualified the professional degree of Doctor of Dental Surgery.

5. In the National University's dental department in the city of Washington, D. C., the diplomas are signed by the President of the United States. This we think originated with General Grant.

6. Almost all the great Universities, including those of the city of Washington, have added dental departments, and are granting professional degrees by the authority of the United States government.

7. The American Medical Association has recognized dentists as specialists in medicine, and the International Medical Congress of the whole civilized world has done the same.

8. Dentists have given to the world the first anæsthetics, both nitrous oxide gas and ether, which Dr. Oliver Wendell Holmes has declared "the greatest boon ever given for humanity." They have also furnished the first splint really satisfactory in the treatment of fractured jaws. This splint was applied in the case of the Hon. W. H. Howard, with entirely satisfactory results, and this after the medical profession had utterly failed to reduce the fracture.

9. Both general and oral surgery are impossible without mechanics, and all operations both in general and oral surgery are more or less mechanical.

10. In the past, both surgery and dentistry were classed among the trades; but time, progress, cultivation, and advancement have altered the condition of things, and to-day surgery has its place on the top round of the medical ladder.

11. The manufacturer makes out of certain materials a new product and offers it on the market for general and indiscriminate use, as for instance the brick-maker.

12. Dentists are not, in the ordinary acceptation of the term, either mechanics or manufacturers, any more than the general surgeon. He applies his manufacture, such as improvising splint or pessary, as the case may require, altering his product as the necessity of the case may demand, and does this through his professional knowledge of anatomy, etc., aided by manipulative ability and mechanical skill. The dentist does precisely the same thing. The oculist adjusts and fits artificial eyes by fitting and grinding as the dentist does artificial teeth.

13. Artificial sets of teeth cannot be offered for sale, giving each customer an opportunity to select what he likes, for no set of teeth could fit, or ever be fitted, for any but the one individual for whom it was especially made; therefore, no one can select a set of teeth as he might a hatchet or hand-saw or any other manufactured article in market.

14. Dental manufacturers are never classed by business men with dentists; we are a separate and distinct body of men.

15. The Census Report of 1880 declares, "That persons following individual pursuits shall be classed together," and we find in that report, headed "persons engaged in selected occupations," lawyers, physicians, dentists, druggists, preachers, etc., classed together. That in the census of 1880 the precedent is established; and as a matter of fact, under instructions from the Superintendent of the Census, General Francis A. Walker, the reports which dentists made in the city of New York were returned to them, and no further attempt was made to classify them as manufacturers, and we respectfully refer you to the correspondence between Dr. Thomas E. Gunning, of New York, and General Walker at that time.

16. The druggist from manufactured ingredients makes a new product to relieve suffering humanity. The dentist does the same thing.

17. We would call your attention to the fact that all the material used by dentists have, or should have, been classed under the head of "dental manufactures." This is true of gold and other metals used in dental practice as well as of instruments, artificial teeth, rubber, etc.; there is no exception to this.

18. Many dental associations have forcibly expressed their sense of the great wrong done to the dental profession by being classed with manufacturers, when no other specialty of medicine is so treated.

We would appeal to your sense of fairness. We claim to be a respectable and law-abiding profession, and ask justice at your hands.

For more than fifty-one years we have worked steadily and faithfully to gain our position and standing. This effort dates from the founding of the first college of dental surgery in the city of Baltimore.

We trust you will see the injustice of deposing us from our honorably-acquired position as professional men, and relegating us to our starting-point. We appeal to you, as officers of this great country to save us from this great wrong, which will undo the work of half a century.

You can readily replace us to where we were in the census of 1880, in the list of "persons engaged in selected occupations." At this time all dental colleges in the United States are raising the standard of culture, and have increased the time required for preparation for graduation from two to three years.

A rigid examination has to be passed in anatomy, physiology, pathology, histology, therapeutics, materia medica, chemistry, and oral surgery before the degree of Doctor of Dental Surgery is conferred. Is any such requirement made of any manufacturer?

Leaving this statement in your hands, we feel confident that, when fully informed of the facts of the case, you will in your wisdom do justice to our profession.

Very respectfully,
Ed. Nelson, D.D.S.,
R. B. Winder, Sr., M.D., D.D.S.,
T. S. Waters, D.D.S.,
A. J. Volck, Ph.D., D.D.S.,
Wm. A. Mills, D.D.S.,
David Genese, D.D.S.,
W. S. Twilley, D.D.S.,
*Committee.*

[In connection with the foregoing, the following circular has been sent us for publication.—Ed.]

Dear Doctor,—The Wilcox bill, making a fine of ten thousand dollars upon any one refusing to answer the questions of the Census Bureau, was passed in the House of Representatives on Saturday, June 4, and will come before the Senate soon. In the census of 1890 the dentists were classed with manufacturers, and as such were presented with similar blanks to fill, which was objected to by the profession generally.

It is the purpose of the several committees appointed by the different State and local societies to proceed to Washington and protest against such classification. The undersigned request the President of each society, in his official capacity, and every individual dentist in the State, to write to Hon. Eugene Hale, the chairman of the Committee on Census Matters, protesting against the position in which the census authorities have placed our profession, and asking that we be excluded from the obligations and penalties of the Wilcox bill.

If you will kindly address a letter to Hon. Eugene Hale, United States Senate, Washington, D. C., we will be obliged.

Very truly yours,

J. D. THOMAS,
L. ASHLEY FAUGHT,
A. W. DEANE,
*Committee.*

[The following is the bill introduced by Hon. Mr. Wilcox. It was referred to the Select Committee on Eleventh Census, March 29, 1892, and reported, with amendments, April 23, and finally passed the House of Representatives, June 4, 1892.—ED.]

### A BILL

### AMENDATORY OF AN ACT ENTITLED "AN ACT TO PROVIDE FOR THE TAKING OF THE ELEVENTH CENSUS."

*Be it enacted by the Senate and House of Representatives of the United States of America in Congress assembled,* That sections fifteen and seventeen of the act entitled "An Act to provide for taking the Eleventh and subsequent censuses," approved March first, eighteen hundred and eighty-nine, be, and the same are, hereby amended so that the Superintendent of Census shall be required to obtain from every incorporated and unincorporated company, firm, ASSOCIATION, *or persons engaged in any productive industry,* the information called for and specified in the general and special schedules heretofore approved *or to be hereafter approved by the Secretary of the Interior.* And every president, treasurer, secretary, agent, director, or other officer of every corporation engaged in such productive industry, and every person, firm, manager, or agent of unincorporated companies, and members of firms, ASSOCIATIONS, *or individuals likewise engaged in such productive industry,* from which or whom answers to any of the inquiries contained in the said schedules are herein required, who shall, if thereto requested by the Superintendent of Census, supervisor, enumerator, or special agent, or each or any of them, wilfully neglect or refuse to give true and complete answers to any inquiry or inquiries contained in the said schedules, or shall wilfully give false information in respect thereto, shall be deemed guilty of a misdemeanor, and on conviction thereof shall be fined in a sum not exceeding ten thousand dollars, to which may be added imprisonment for a period not exceeding one year. And all acts or parts of acts in conflict herewith are hereby repealed.

---

### MEETING OF THE PENNSYLVANIA STATE DENTAL SOCIETY AND THE JOINT MEETING OF THE PENNSYLVANIA AND THE NEW JERSEY STATE DENTAL SOCIETIES.

THE business meeting of the Pennsylvania State Dental Society occurs on Wednesday, July 20, at 10 o'clock A.M., at Cresson, Pa. Those attending should leave Philadelphia not later than the 11.20 P.M. train of Tuesday.

The joint meeting of the two Societies will commence on Thursday, July 21, at 10 A.M., beginning with the addresses of the presidents of each Society, followed by four papers, to each of which two formal replies will be read.

A varied programme of papers, clinics, and a clinical conference is provided for, all of which make promise of an interesting meeting.

H. NEWTON YOUNG,
*Corresponding Secretary Penna. State Society.*

---

### AMERICAN DENTAL ASSOCIATION.

THE Thirty-second Annual Session of the American Dental Association will be held at Niagara Falls, New York, commencing at 10 o'clock A.M., Tuesday, August 2, 1892.

GEO. H. CUSHING,
*Recording Secretary.*

---

### NEW JERSEY EXAMINATIONS.

THE next meeting of the New Jersey State Board of Examiners will be held at the Commission Rooms, No. 88 Broad Street, Elizabeth, N. J., on Monday and Tuesday, July 18 and 19. Candidates will please file their applications with the secretary before July 5. Blanks and information furnished on application.

G. Carleton Brown,
*Secretary.*

88 Broad Street, Elizabeth, N. J.

---

### NATIONAL ASSOCIATION OF DENTAL FACULTIES.

The Ninth Annual Session of the National Association of Dental Faculties will be held at Niagara Falls, commencing Monday, August 1, 1892, at 10 o'clock A.M.

Colleges making application for membership must send the same to Dr. J. Taft, sixty days before the time of meeting.

J. D. Patterson,
*Secretary.*

May 20, 1892.

---

### FIRST DISTRICT DENTAL SOCIETY, STATE OF NEW YORK.

At the Annual Meeting of the First District Dental Society of the State of New York, held April 12, 1892, the officers elected for the ensuing year were as follows:

President, William Carr; Vice-President, John I. Hart; Secretary, Benj. C. Nash; Treasurer, Kasson C. Gibson; Librarian, J. Bond Littig. Delegates to the Dental Society of the State of New York, for four years, J. W. Taylor and William C. Deane; for one year, Henry J. Hull.

Standing Committees appointed by the President: Executive Committee, A. L. Northrop, Delos Palmer, and William Wallace Walker, chairman. Clinic Committee, John H. Meyer, Henry J. Hull, and C. F. W. Bödecker, chairman.

B. C. Nash,
*Secretary.*

---

### MINNESOTA STATE DENTAL ASSOCIATION.

The Minnesota State Dental Association will hold its annual meeting July 13, 14, and 15, at Minneapolis.

L. D. Leonard,
*Secretary.*

251 Nicollet Avenue, Minneapolis, Minn.

### SOUTH CAROLINA STATE DENTAL ASSOCIATION

THE Twenty-second Annual Meeting of the South Carolina State Dental Association, and of the State Board of Dental Examiners, will be held at Rock Hill, S. C., commencing on Tuesday, July 12, 1892, at 10 o'clock A.M.

Officers: President, J. T. Calvert; First Vice-President, C. S. Patrick; Second Vice-President, Theo. Johnston; Corresponding Secretary, A. T. Peete; Recording Secretary, B. Rutledge; Treasurer, G. W. Dick.

---

### PATENTS.

A LIST of recent patents, reported specially for the INTERNATIONAL DENTAL JOURNAL.

472,344. Metal Tooth-Crown. George Evans, New York, N. Y. Filed September 7, 1891.

Trade Mark. 20,929. Aluminum Solder for dental Plates and other uses. Charles Gerard and Joseph S. Letord, Kansas City, Mo. Filed March 1, 1892.

472,683. Coupling for dental engine shafts and hand-pieces. Johannes Th. Pedersen, New York, N. Y., assignor to William E. Wells, same place. Filed August 25, 1891.

472,686. Dental Mallet. William E. Wells, New York, N. Y. Filed August 24, 1891.

472,776. Dental Engine. Wallace W. Williamson, Syracuse, N. Y. Filed May 28, 1891.

473,040. Teeth Regulator. Deel R. Wilder, Los Angeles, Cal. Filed February 2, 1892.

474,011. Dental Tool. Almer M. Harrison, Columbus, Ohio. Filed August 28, 1891.

474,104. Artificial Denture. John J. Stedman, La Porte, Ind. Filed December 21, 1891.

474,131. Dental Cervix-Clamp. James W. Ivory, Philadelphia, Pa. Filed August 2, 1890.

474,132. Dental Matrix-Retainer. James W. Ivory, Philadelphia, Pa. Filed May 11, 1891.

474,967. Dental Process. Joseph Payne, Dwight, Ill. Filed February 9, 1891.

474,968. Dental Articulator. Joseph Payne, Dwight, Ill. Filed February 9, 1891.

475,141. Artificial Tooth. David B. McHenry, Grenada, Miss. Filed December 21, 1891.

### CONNECTICUT STATE DENTAL ASSOCIATION.

THE Twenty-eighth Annual Meeting of the Connecticut State Dental Association was held at Hartford, Tuesday, May 17. A pleasant session closed with the annual dinner at "The Henblein." The following officers were re-elected :

Dr. E. S. Gaylord, of New Haven, President; Dr. C. C. Barker, of Meriden, Vice-President; Dr. George L. Parmele, of Hartford, Secretary ; Dr. Joseph H. Smith, of New Haven, Treasurer.

*Executive Committee.*—Dr. James McManus, Hartford; Dr. William H. Rider, Danbury; Dr. Daniel A. Jones, New Haven.

G. L. PARMELE,
*Secretary.*

---

### ILLINOIS STATE DENTAL SOCIETY.

THE Twenty-eighth Annual Meeting of the Illinois State Dental Society was held at Springfield, May 10–13, 1892. The following officers were elected for the ensuing year:

President, E. K. Blair, Waverly; Vice-President, N. Johnson, Chicago; Secretary, Louis Ottofy, Chicago; Treasurer, W. A. Stevens, Chicago; Librarian, F. H. McIntosh, Bloomington.

The next meeting will be held at Rock Island, second Tuesday in May, 1893.

LOUIS OTTOFY,
*Secretary.*

MASONIC TEMPLE, CHICAGO.

---

### BUFFALO DENTAL ASSOCIATION.

AT a meeting of the Buffalo Dental Association, called for the purpose of considering the action of the University of Buffalo in conferring honorary degrees in dentistry, the enclosed preamble and resolutions were adopted with but one dissenting voice.

"WHEREAS, The dental colleges of the United States, recognizing the needs of the profession, banded themselves together in 1884 in a voluntary association known as 'The National Association of Dental Faculties,' for the purpose of raising the standards in all matters pertaining to dental education ; and,

"WHEREAS, In order to reach this end they surrendered a right secured to them by their charter and long exercised by them, the right to confer 'honorarily' the degree of Doctor of Dental Surgery; and,

"WHEREAS, This voluntary action on the part of the colleges met with the hearty approval and support of the whole dental profession; therefore

"*Resolved*, That we, 'The Buffalo Dental Association,' believing it to be the duty of the profession to uphold and sustain the colleges in every effort for the uplifting of the standards of dental education, desire to express our unqualified disapproval of the granting of honorary degrees in dentistry under any and all circumstances, but more particularly when it is done, as in the recent action of the 'University of Buffalo,' for the express purpose of fitting men to hold positions in the Faculty of a proposed dental department.

"*Resolved*, That we earnestly ask the profession to so rebuke this action of the 'University of Buffalo' as to make it certain that hereafter no university will dare do that which no dental college can do without losing its standing in the dental profession."

W. C. HAYES, M.D.S., *President*.
RICHARD KESSELL, D.D.S., *Secretary*.

---

### COMMENCEMENT.

### UNIVERSITY OF PENNSYLVANIA—DENTAL DEPARTMENT.

| | |
|---|---|
| Matriculates | 169 |
| Graduates June, 1891 | 8 |
| Graduates May 6, 1892 | 84 |
| | 92 |

